# The interdependence of transcript and protein abundance: new data–new complexities

**DOI:** 10.15252/msb.20156720

**Published:** 2016-01-20

**Authors:** Yansheng Liu, Ruedi Aebersold

**Affiliations:** ^1^ Department of Biology Institute of Molecular Systems Biology ETH Zurich Zurich Switzerland; ^2^ Faculty of Science University of Zurich Zurich Switzerland

**Keywords:** Genome-Scale & Integrative Biology, Membrane & Intracellular Transport

## Abstract

The relative contribution of transcriptional and translational regulation in gene expression control has been intensely debated and remains a challenging question. Recent reports have suggested that protein abundance in mammalian cells is primarily controlled at the transcript‐level. In their recent work, Cheng *et al* (2016) determined the proteomic and transcriptomic changes in cells responding to endoplasmic reticulum (ER) stress. Their analyses indicate that the ER stress response is significantly controlled at both the transcript and protein levels.

The central dogma of biology describes the flow of information from gene to transcript to protein, but it makes no statement about the relationship between transcript and protein levels, or the mechanisms that control them (Vogel & Marcotte, 2012; McManus *et al*, 2015). Gene expression adapts protein levels to specific cellular states and is controlled at the transcriptional, translational, and post‐translational levels. Understanding the relative contribution of these different levels that collectively control protein abundance as a function of cellular state is important for basic and translational science, for example, for predicting protein levels in response to genomic or epigenomic variability or altered environmental conditions.

Recent studies have suggested that changes in mRNA levels strongly determine protein dynamics in many scenarios (Li & Biggin, 2015). For example, Li *et al* re‐analyzed data generated in exponentially growing, non‐synchronized mouse embryonic fibroblasts (Schwanhausser *et al*, 2011) and found that mRNA abundance determined 84% of the gene‐to‐gene differences at the protein level, whereas protein synthesis and degradation, respectively, only determined about 8% (Li *et al*, 2014). More recently, Jovanovic *et al* analyzed the kinetics of protein and mRNA expression in mouse bone marrow‐derived dendritic cells following lipopolysaccharide (LPS) stimulation. They concluded that in unstimulated cells nearly two‐thirds of the gene‐to‐gene variation in total protein levels was explained by mRNA regulation. Moreover, they found that during the response to LPS, the fold changes of the majority of the proteome were still dominated by mRNA abundance dynamics, whereas the preexisting proteome performing basic functions was primarily regulated through protein production and degradation (Jovanovic *et al*, 2015).

In their recent work, Cheng *et al* (2016) extended this line of research to a third experimental system. They studied the dynamics of transcript and protein profiles in HeLa cells following induction of ER stress by dithiothreitol (DTT) treatment. Interestingly, their results argue for an approximately equal weight of the protein‐ and mRNA‐level responses to establishing the observed changes in the dynamic proteome profiles, thus suggesting that the relative contribution of mRNA‐ and protein‐level regulations in the overall gene expression scheme is dependent on the biological system.

The experimental system used by Cheng *et al* (2016) is characterized by the severity of induced stress. Significant apoptosis was observed, peaking at 2 h after DTT‐induced ER stress, at which point ~45% of the cells had undergone cell death. ER stress is known to activate the unfolded protein response (UPR) that can restore normal cellular function in stressed cells by halting protein translation, removing the misfolded proteins and re‐arranging the cellular proteome. Therefore, the system of severe ER stress investigated by Cheng *et al* (2016) induces a vital, substantial, and pleiotropic cellular response, a situation that is in stark contrast to cells in steady state or after the stimulation of specific signaling systems (Fig 1). In comparison, the LPS stimulation of dendritic cells seems to trigger a substantially milder burden to the cells with a much lower fraction of cell death being apparent (personal communication with Marko Jovanovic) (Jovanovic *et al*, 2015). Importantly, in both studies, the molecular analyses were performed on cells surviving the stress, while cell debris was discarded.

**Figure 1:**
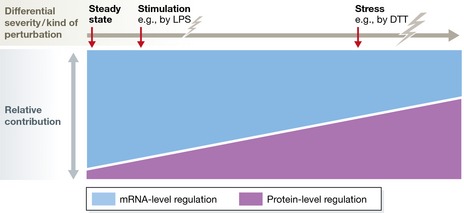
The relative contribution of mRNA‐level (transcriptional control) and protein‐level (protein synthesis and degradation control) regulations is context dependent Based on the data from Jovanovic *et al* (2015), Cheng *et al* (2016) and previous studies on steady‐state measurements (Schwanhausser *et al*, 2011; Vogel & Marcotte, 2012), the relative contribution of translational processes controlling protein abundance seems to increase with the increasing severity of stress and to depend on the kind of perturbation applied. LPS, lipopolysaccharide; DTT, dithiothreitol.

Time‐resolved measurement of protein and transcript levels following stress induction can reveal concordant and discordant changes between mRNA and protein profiles for each transcript/protein pair (Fournier *et al*, 2010; Jovanovic *et al*, 2015). The study of Cheng *et al* (2016) was performed over a 30 h of time course, at seven time points including time point zero. This design allowed the authors to apply their previously established PECA software (Teo *et al*, 2014) to analyze the extensive dataset. PECA is the first computational method to analyze the ratio of synthesis and degradation rates over successive time intervals. It determines regulatory events based on the significance of the changes of the respective rates between time points, in a statistically consistent manner. (Teo *et al*, 2014). Interestingly, visualization of the data as a simple heatmap of RNA and protein synthesis and degradation rates computed by PECA indicates that the time‐resolved ER responses at the transcript and protein level differed substantially. RNA‐level regulation showed a “spike‐like” behavior during the time course and generally returned to prestress levels within the time‐course tested. In contrast, protein‐level regulation for the most part followed a “switch‐like” pattern, whereby a major fraction of the proteome was quantitatively remodeled to reach a new steady state. As the authors correctly point out, their study lacks transcript data for the earliest 30 min following stress, and therefore, further studies are needed to also resolve the regulatory pattern at these early time points.

What is the relative contribution of mRNA‐level versus protein‐level regulation in this ER stress system? First, the authors addressed this question by comparing the magnitude of fold change for mRNAs and proteins, respectively, and found significantly more pronounced fold changes at the protein level. Second, similar numbers of each molecular species were identified as significantly changed demonstrating that mRNA‐ and protein‐level changes are similarly prevalent in their system. Although the authors did not quantify the contribution of transcriptional, translational, and degradation processes to the observed overall protein variance, their data compellingly illustrate the large difference between protein and RNA dynamics in terms of amplitude and temporal profiles.

The work of Cheng *et al* (2016) also raises several questions that remain to be answered by future studies. For example, the re‐analysis of data from Jovanovic *et al* by the methods used in this work suggests that LPS stimulation, in contrast to DTT‐induced ER stress, triggered a “switch‐like” regulation at the RNA, but not at the protein level. Some relevant questions include the following: Does the switch‐like pattern always reflect the prevalent regulatory level and directly lead to a new steady state in any system? For how long does the new proteomic state remain stable after DTT degradation? Are there dynamic systems in which protein synthesis and degradation processes override transcriptional regulation?

To summarize, the data generated by Cheng *et al* (2016), examining the interdependence of mRNA and protein levels in a system responding to ER stress, together with previous studies, indicate that the relative contribution of mRNA‐ versus protein‐level regulation seems to be dependent on the temporal scale, on the complexity level of the biological system and on the type of perturbation applied (e.g., steady states, stimulations of specific signaling systems and severe, pleiotropic stress conditions, as illustrated in Fig 1). The debate on the topic will therefore likely continue.
